# Supplementary material for: Facile Synthesis of B/P Co-Doping Multicolor Emissive Carbon Dots Derived from Phenylenediamine Isomers and Their Application in Anticounterfeiting
Source: Nanomaterials (Basel). 2024 May 7;14(10):813. doi: 10.3390/nano14100813 (PMC11123944; doi:10.3390/nano14100813)
Supplement: Supplementary file 1 [file nanomaterials-14-00813-s001.zip › nanomaterials-2988932-supplementary.pdf]

nanomaterials

Article

Facile Synthesis of B/P Co-Doping Multicolor Emissive Carbon Dots Derived from Phenylenediamine Isomers and Their Application in Anticounterfeiting

Zhiwei Li [1,2]

[1] Key Laboratory for Polymeric Composite and Functional Materials of Ministry of Education, School of Chemistry, Sun Yat-sen University, Guangzhou 510275, China; lizhw36@mail2.sysu.edu.cn
[2] State Key Laboratory of Optoelectronic Materials and Technologies, Sun Yat-sen University, Guangzhou 510275, China

Abstract: Carbon dots (CDs) possess a considerable number of beneficial features for latent applications in biotargeted drugs, electronic transistors, and encrypted information. The synthesis of fluorescent carbon dots has become a trend in contemporary research, especially in the field of controllable multicolor fluorescent carbon dots. In this study, an elementary one-step hydrothermal method was employed to synthesize the multicolor fluorescent carbon dots by co-doping unique phenylenediamine isomers (o-PD, m-PD, and p-PD) with B and P elements, which under 365 nm UV light exhibited signs of lavender-color, grass-color, and tangerine-color fluorescence, respectively. Further investigations reveal the distinctness in the polymerization, surface-specific functional groups, and graphite N content of the multicolor CDs, which may be the chief factor regarding the different optical behaviors of the multicolor CDs. This new work offers a route for the exploration of multicolor CDs using B/P co-doping and suggests great potential in the field of optical materials, important information encryption, and commercial anticounterfeiting labels.

Keywords: phenylenediamine; isomer; multicolor CDs; co-doping; anticounterfeiting

Citation: Li, Z. Facile Synthesis of B/P Co-Doping Multicolor Emissive Carbon Dots Derived from Phenylenediamine Isomers and Their Application in Anticounterfeiting. *Nanomaterials* **2024**, *14*, 813. https://doi.org/10.3390/nano14100813

Academic Editor: Michael Tiemann

Received: 15 April 2024
Revised: 3 May 2024
Accepted: 4 May 2024
Published: 7 May 2024

Copyright: © 2024 by the author. Licensee MDPI, Basel, Switzerland. This article is an open access article distributed under the terms and conditions of the Creative Commons Attribution (CC BY) license (https://creativecommons.org/licenses/by/4.0/).

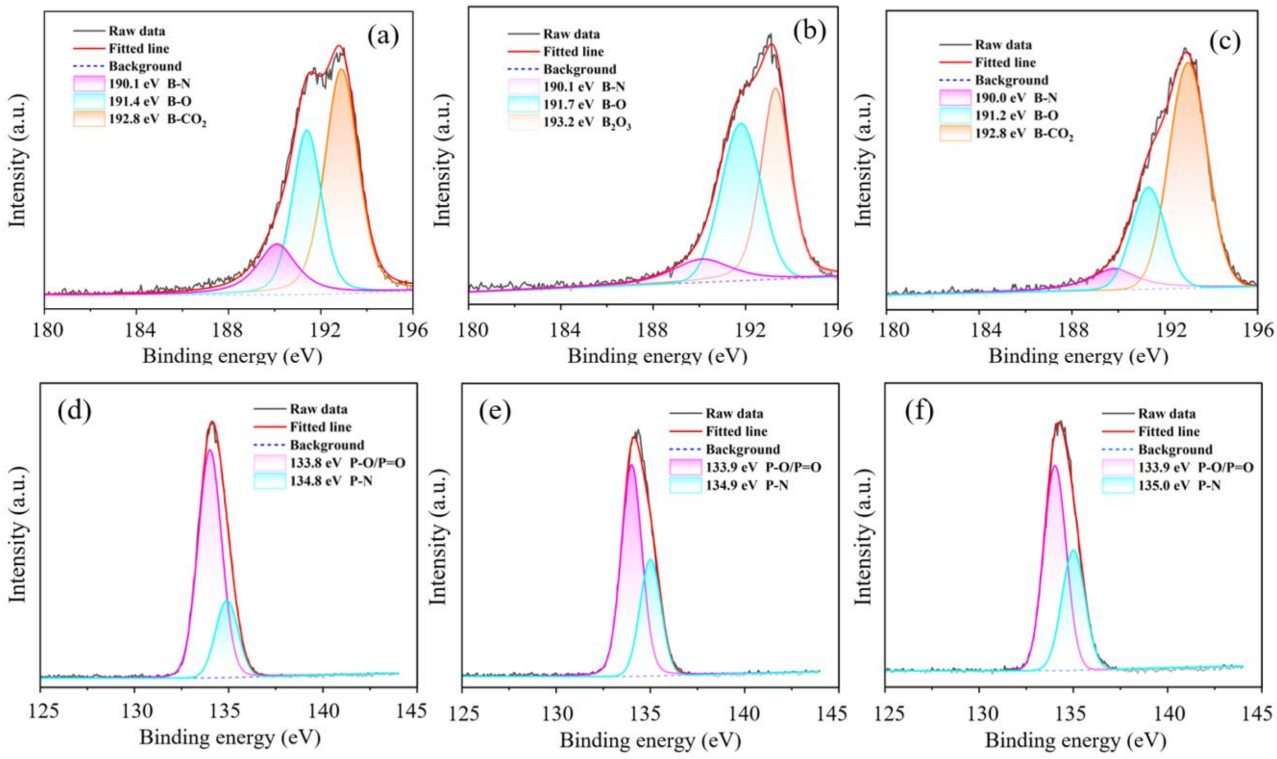

Figure S1. (a–c) High-resolution XPS spectra of B 1s for L-CDs, G-CDs and T-CDs. (d-f) High-resolution XPS spectra of P 2p for L-CDs, G-CDs and T-CDs.

Table S1. Specific optical parameters of the multicolor CDs.

sample	L-CDs	G-CDs	T-CDs
Ex/Em (nm)	344/414	458/515	537/619
molar absorption coefficients ($cm^{-1}M^{-1}$)	558	459	472
fluorescence quantum yield	5.2 %	6.0 %	4.5 %
fluorescent brightness	29.02	27.54	21.24

Disclaimer/Publisher's Note: The statements, opinions and data contained in all publications are solely those of the individual author(s) and contributor(s) and not of MDPI and/or the editor(s). MDPI and/or the editor(s) disclaim responsibility for any injury to people or property resulting from any ideas, methods, instructions or products referred to in the content.
